# Stability of Betaine Capsules

**DOI:** 10.1155/2013/458625

**Published:** 2013-06-03

**Authors:** Mirza Akram Hossain, Stéphanie Boily, Natasha Beauregard, Jean-Marc Forest, Grégoire Leclair

**Affiliations:** ^1^Faculty of Pharmacy, Université de Montréal, P.O. Box 6128, Downtown Station, Montreal, QC, Canada H3C 3J7; ^2^CHU Ste-Justine, 3175, ch. de la Côte-Sainte-Catherine, Montreal, QC, Canada H3T 1C5

## Abstract

Betaine is used to treat homocystinuria and is not available in Canada as a formulated drug. In order to facilitate the administration of this compound to patients, a capsule formulation and an evaluation of its stability were required. Capsule formulations of betaine were developed (160 mg and 625 mg of betaine per capsule). As betaine has no chromophore, an HPLC-ELSD analytical method was also developed. The critical quality attributes of these formulations were evaluated (content assay, content uniformity, and dissolution) as well as their stability. Capsules with acceptable quality attributes were produced. These capsules remained stable for 1 year when stored in airtight containers at controlled room temperature. However, shelf life decreased dramatically in nonairtight containers at 30°C (3 months for the lactose-containing capsules of 160 mg and 6 months for the capsules of 625 mg).

## 1. Introduction

Homocystinuria is a metabolic disorder that appears in early childhood and is characterized by homocysteine excess in the blood stream. Symptoms of homocystinuria are multiple and include severe growth and bone impairments. Patients must have lifetime follow-ups by healthcare providers in order to keep their condition under control [1–4].

Betaine is used to treat homocystinuria and is commercialized as a white granulated powder that can be dissolved in 120 to 180 mL of water, juice or milk and administered immediately [1–5].

The dosage of betaine should be between 100 and 150 mg/kg/day in 2, 3, or 4 doses. The exact dose is calculated from the concentration of homocysteine in the blood. Betaine doses required to treat each patient must therefore be individualized [1]. Betaine in gelatin capsules facilitates administration by preventing patients from having to measure or weigh their medication. They can either swallow the capsule or open it and dissolve its content in the appropriate vehicle. Although betaine in capsule compounded formulations are prepared by hospital pharmacists in Canada, the stability of this preparation has not been previously reported.

**Figure 1:**
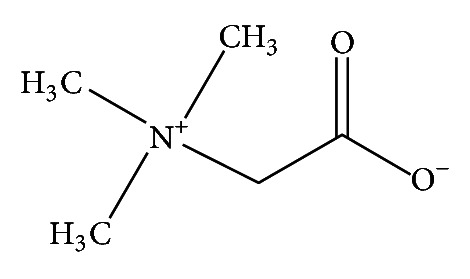
Chemical structure of betaine.

Betaine, also known as betaine oxyneurine, or trimethylglycine, is an inner salt formed from a quaternary ammonium and a carboxylate (Figure 1). This crystalline powder is very soluble in water (160 g/100 g of water) and is also hygroscopic [6]. Based on the chemical structure of this compound, chemical stability is not expected to be problematic under nonenzymatic conditions. However, a monohydrated form has been reported, and therefore solubility could be affected by changes in the solid phase [6].

In order to evaluate the stability of capsule preparations of betaine, it is important to first identify the critical quality attributes of this formulation: content uniformity, content assay, and dissolution. Content uniformity can be evaluated by gravimetric analysis if betaine levels are at least 25% of the total content of the capsules [7]. However, assay and dissolution will require an adequate analytical method. Because of its very high polarity, betaine is not retained by reverse phase chromatography. In addition, betaine has no chromophore and cannot be quantified using a UV detector. Because of those properties, a novel chromatographic method is needed to be developed. Normal phase chromatography coupled to an evaporative light scattering detector could then be considered.

The objectives of this study are as follows (1) To develop an analytical method to quantify betaine in aqueous solutions; (2) to define a laboratory-scale manufacturing process to prepare high dose and low dose betaine; and (3) to evaluate the stability of betaine capsules under different environmental conditions.

## 2. Materials and Methods

### 2.1. Materials

Capsules were prepared with a BB-3/S capsule filler equipped with an AB-4/7 capsule sorter (Dott. Bonapace & Co., Limbiate, MB, Italy). Transparent size 00 gelatin capsules (product 3901-2001, lot 10421-0063), lactose monohydrate NF (product LA530, lot 10069-0097), and betaine anhydrous (product BE391, lot 10273-0273) were purchased from Galenova, St-Hyacinthe, QC, Canada. Betaine anhydrous (product B2629, lot 070M1865V) was also purchased from Sigma-Aldrich, St. Louis, MS, USA, in order to evaluate a second supplier.

### 2.2. Preparation and Incubation of the Capsules

Capsules containing 160 mg and 625 mg of betaine were prepared using a semiautomatic capsule filler system. The 625 mg capsules were prepared by direct encapsulation of pure betaine (93.75 g) in size 00 gelatin capsules (150 capsules per tray). The 160 mg capsules were prepared by first mixing betaine (24 g) with lactose monohydrate (72 g) using a mortar and a pestle. The blend was then encapsulated into size 00 gelatin capsule (150 capsules per tray).

A total of six lots of 750 capsules were prepared. Lots 160A, 160B, 625A, and 625B were prepared from betaine sourced from Sigma-Aldrich (German origin). Lots 160C and 625C were prepared from betaine sourced from Galenova (Chinese origin).

Each lot was packaged in snap cap PE amber vials (17 capsules/vials) as well as doubled Ziploc^tm^ bags (120 capsules/bag) and then stored at 22°C/ambient RH (3, 6, 9, and 12 months), 30°C/65% RH (3, 6, 9, and 12 months), and 40°C/75% RH (1, 2, 3, and 6 months).

### 2.3. Chromatographic Method

Betaine was quantified with high performance liquid chromatography (HPLC) using a Shimadzu Prominence UFLC system equipped with an evaporative light scattering detector (ELSD, Shimadzu ELSD-LTII). A normal-phase silica column (Luna 3u, Phenomenex, 100 × 2.0 mm) was used with the matching silica precolumn (4 × 2 mm). The mobile phase consisted of a 86 : 14 (v/v) mixture of acetonitrile and 1% ammonium acetate in water. The flow rate was 1 mL/min for 6 min, and the column temperature was set at 40°C. The injection volume used was 5 *μ*L. The ELSD detector nebulizer heater was set at 80°C with a gain of 7. The nitrogen pressure was maintained at 50 psi.

For evaporative detectors like the ELSD, the response signal is typically an exponential function of the concentration of the analyte. Therefore, linearity of the method was verified after logarithmic transformation of the concentrations and the response signal (ln⁡*C* = *a*ln⁡*R* + *b*, where *C* is the concentration, *R* is the response signal, and *a* and *b* are the fitting parameters). Linearity was evaluated between 0.14 and 0.84 mg/mL (5 points, *r* ^2^ = 0.9999, used for the analysis of 625 mg capsules) and also between 0.04 and 0.22 (5 points, *r* ^2^ = 0.999, used for the analysis of 160 mg capsules). Gelatin, lactose, and betaine solutions were separately analysed and then coinjected to confirm the specificity of the method. No overlapping peaks were observed.

### 2.4. Uniformity of Dosage Units

Uniformity of dosage units was evaluated according to USP <905> using the weight variation method for hard capsules. This evaluation was performed for each lot after manufacturing. Maximum allowed acceptance values of 15.0 (L1, 10 capsules) and 25.0 (L2, 30 capsules) were used as recommended by the USP [7].

### 2.5. Visual Appearance

All capsules were visually examined to identify any evidence of gross degradation or loss of function after manufacturing. The description specification of both 160 mg and 625 mg capsules was as follows: “size 00 transparent capsule filled with off-white powder.” Similarly, this examination was performed on all pulled out capsules from stability stations.

### 2.6. Dissolution and Content Assay

Dissolution was performed in aqueous hydrochloric acid (0.1 N, 900 mL, 37°C) using an USP apparatus II equipped with paddles (75 rpm). Capsules were weighted using capsule sinkers to avoid buoyancy. Dissolutions were replicated 6 times, and samples were taken every 15 min until capsule shell and content were completely dissolved. Samples were then analysed by HPLC. The dissolution medium at the final time point was a clear solution and was considered representative of the total content of the capsule.

Dissolution of not less than 85% after 15 min and content assay between 90% and 110% were considered acceptable for this immediate release formulation. In addition, the content assay of stability samples was evaluated and compared to the initial samples. A content assay variation of not more than 10% relative to the initial value was considered acceptable. This test was performed for each manufactured lot and also pulled out capsules from the stability station. The test was not performed when the capsules already failed visual examination.

## 3. Results and Discussion

### 3.1. Uniformity of Dosage Units

The uniformity of dosage units was verified by gravimetric analysis. The acceptance value (AV) was calculated for each lot as per USP <905>. The test was first performed using 10 capsules to calculate the acceptance value. As shown in Table 1, the acceptance value was not more than 15.0 for each lot, and no further testing was required. In order to illustrate the scattering of the individual capsule content, the minimal and maximal individual contents are listed along with the average content of units and its relative standard deviation. Each lot complied with the USP <905> specifications for uniformity of dosage units.

### 3.2. Visual Examination

All capsules complied with the description specification after manufacturing. However, presence of lactose, packaging, and environmental conditions dramatically impacted the visual appearance of the capsules. Indeed, capsule browning, deformation, and even liquefaction were observed during the stability studies.  Table 2 illustrates the last time point where the capsules passed the visual examination test and the first time point where it failed the same test. Presence of lactose in the 160 mg capsules was slightly detrimental to its stability when stability results in airtight bags are compared. The most important factor was humidity, as packaging in nonairtight vials significantly reduced the stability of the capsules. From the visual examination results, it is obvious that these capsules need to be stored in airtight packaging and protected from heat.

### 3.3. Content Assay and Dissolution

As previously mentioned, the capsules were analyzed for content and dissolution only if they had passed the visual examination. As shown in Table 3, all analyzed capsules complied with the dissolution and the content assay specifications.

## 4. Conclusion

Betaine capsules need to be protected from heat and moisture. Adequate packaging should be airtight. When protected from moisture and stored at controlled room temperature, these capsules are stable for at least 1 year. After dispensing, the patient should be advised to keep the capsules protected from moisture and heat (airtight container and below 30°C) for a period no longer than 3 months for lactose-containing capsule and 6 months for pure betaine capsules.

## Figures and Tables

**Table 1:** Uniformity of dosage units.

	Lot 160A	Lot 160B	Lot 160C	Lot 625A	Lot 625B	Lot 625C
Average (%)	103.9	107.5	102.4	103.2	103.8	105.1
Min (%)	101.1	103.5	99.4	98.9	99.3	103.1
Max (%)	107.4	112.1	106.6	106.6	105.9	108.0
RSD (%)	1.7	2.3	2.2	2.3	1.9	1.3
AV	6.6	11.6	6.2	7.2	7.0	6.6

RSD: relative standard deviation; AV: acceptance value.

**Table 2:** Visual examination of betaine capsules.

	160 mg bags	160 mg vials	625 mg bags	625 mg vials
22°C				
Pass	12 months	3 months	12 months	3 months
Fail	—	6 months	—	6 months
30°C/65% RH				
Pass	3 months	Initial	6 months	Initial
Fail	6 months	3 months	9 months	3 months
40°C/75% RH				
Pass	Initial	Initial	2 months	Initial
Fail	1 month	1 month	3 months	1 month

**Table 3:** Content assay and dissolution of the betaine capsules.

	Initial (mg)	1 month (%)	2 months (%)	3 months (%)	6 months (%)	9 months (%)	12 months (%)
22°C—ambient RH—vials							
160 mg	167 ± 4	NT	NT	102.5 ± 1.6	F	F	F
625 mg	650 ± 6	NT	NT	100.5 ± 1.5	F	F	F
30°C—65% RH—vials							
160 mg	167 ± 4	NT	NT	F	F	F	F
625 mg	650 ± 6	NT	NT	F	F	F	F
40°C—75% RH—vials							
160 mg	167 ± 4	F	F	F	F	F	F
625 mg	650 ± 6	F	F	F	F	F	F
22°C—ambient RH—bags							
160 mg	167 ± 4	NT	NT	102.2 ± 5.8	96.7 ± 3.3	94.3 ± 0.1*	93.4 ± 0.4*
625 mg	650 ± 6	NT	NT	98.3 ± 1.9	103.2 ± 0.7	98.2 ± 0.8	96.9 ± 0.5
30°C—65% RH—bags							
160 mg	167 ± 4	NT	NT	102.1 ± 4.0	F	F	F
625 mg	650 ± 6	NT	NT	100.6 ± 1.1	100.0 ± 0.5	F	F
40°C—75% RH—Bags							
160 mg	167 ± 4	F	F	F	F	F	F
625 mg	650 ± 6	98.2 ± 2.2	100.1 ± 1.6	F	F	F	F

Initial results are reported in mg/capsule; stability results are reported as percentage of initial value; unless otherwise noted, all capsules completely dissolved within 15 min; *for 1 capsule out of 3 × 6 replicates, a few undissolved particulate fragments remained after 15 min and these fragments completely dissolved within 30 min. The 15 min release was not less than 85%; F: failed visual examination; NT: not tested.
